# Private screen access in early adolescence predicts subsequent academic and social impairment at the end of high school for boys and girls

**DOI:** 10.24095/hpcdp.44.2.01

**Published:** 2024-02

**Authors:** Benoit Gauthier, Linda S. Pagani

**Affiliations:** 1 Faculty of Arts and Science, Universit de Montral, Montral, Quebec, Canada; 2 School of Psychoeducation, Universit de Montral, Montral, Quebec, Canada; 3 Centre hospitalier universitaire Sainte-Justine, Universit de Montral, Montral, Quebec, Canada; 4 School Environment Research Group, Universit de Montral, Montral, Quebec, Canada

**Keywords:** bedroom screens, private access, adolescent health, adolescent development, academic adjustment, social adjustment

## Abstract

**Introduction:**

Youth media guidelines in Canada and the United States recommend that bedrooms should remain screen-free zones. This study aims to verify whether bedroom screens at age 12 years prospectively predict academic and social impairment by age 17years.

**Methods:**

Participants were from the Quebec Longitudinal Study of Child Development birth cohort (661 girls and 686 boys). Linear regression analyses estimated associations between having a bedroom screen (television or computer) at age 12 years and self-reported overall grades, dropout risk, prosocial behaviour and likelihood of having experienced a dating relationship in the past 12 months at age 17 years, while adjusting for potential individual and family confounding factors.

**Results:**

For both girls and boys, bedroom screens at age 12 years predicted lower overall grades (*B*=−2.41, *p*≤0.001 for boys; −1.61, *p*≤0.05 for girls), higher dropout risk (*B*=0.16, *p*≤0.001 for boys; 0.17, *p*≤0.001 for girls) and lower likelihood of having experienced a dating relationship (*B*=−0.13, *p*≤0.001 for boys; −0.18, *p*≤0.001 for girls) at age 17. Bedroom screens also predicted lower levels of prosocial behaviour (*B*=−0.52, *p*≤0.001) at age 17 years for boys.

**Conclusion:**

The bedroom as an early adolescent screen-based zone does not predict long-term positive health and well-being. Pediatric recommendations to parents and youth should be more resolute about bedrooms being screen-free zones and about unlimited access in private exposures in childhood.

**Highlights**

- Previous research has shown, and child and adolescent media guidelines recommend, that screens should be kept out of private zones. Early childhood bedroom screen exposure is associated with developmental and health risks, including slower language acquisition, lower sociability and emotional distress in later childhood.
- Using a prospective-longitudinal birth cohort of 661 girls and 686 boys born at a time when screen exposure was less complex, we found that having a bedroom television or computer in early adolescence predicted academic and social risks in later adolescence, likely from overexposure in terms of time and content.

## Introduction

School-aged youth screen exposure has increased in recent years.^1^ Technology is rapidly evolving, and with the burgeoning emergence of portable devices, the times and spaces in which youth can use screen media are multiplying. Most children and teens now spend more than twice the time recommended for daily exposure to leisure screen media.^2^ Guidelines have been established by the American Academy of Pediatrics (AAP) and Canadian Pediatric Society (CPS). These commonly recommend no exposure prior to age 2 years, less than one hour before age 5 years and less than two hours for school-aged youth.^3^,^4^ From middle school onward, the time spent on screens creates a time debt for other enriching developmental activities that shape human capital prospects for social and occupational functioning.^5^

AAP and CPS media-use guidelines also advise parents and youth to designate media-free zones at home.^3^,^4^ Screen placement in a private space such as the bedroom creates solitary and unsupervised accessibility.^6^ With private access, children are more likely to socialize in person less and to study less.^7^

Previous studies have established associations between childhood bedroom screen access and increased overall screen time.^1^,^7^,^8^ In fact, private screen access is associated with greater time spent in one’s bedroom, and thus more isolation and greater screen use.^9^ Recent studies and literature reviews of both cross-sectional and longitudinal studies of children and adolescents of varying ages highlight links between different types of screen exposure, lower levels of prosocial behaviour and less optimal academic performance.^10^-^12^ Compared with those having no screens at all, children and adolescents who have a television, computer or video game system in their bedroom are at higher risk of adiposity and inadequate sleep.^6^,^13^,^14^ However, not much is known about the link between bedroom media in adolescence and social and academic functioning. 

Both the time spent viewing screens and the content viewed may have an impact on children’s development and later lives. According to the time displacement hypothesis, time spent on screens represents time not invested in other, more enriching activities, such as in-person socializing and doing homework.^7^,^15^,^16^ The content hypothesis states that exposure to violent and inappropriate content, which may increase with private screen access, impairs the development of prosocial behaviour.^7^ The ability to empathize is a crucial socioemotional skill that promotes creation of positive social ties.^17^ Engaging in a dating relationship also represents an important developmental task of adolescence.^18^ Finally, graduating from high school represents an important milestone as well, and thus represents a pillar for later social and economic success.^18^-^20^

Some of the existing literature on screen exposure and youth development contains some methodological challenges that weaken interpretations.^21^ First, the risks associated with private screen access in early adolescence have scarcely been examined, and its relationship with social and academic functioning in later adolescence remains unclear. Private access emerges as a measure that potentially provides more information about the nature of content and experiences of teenagers with screens, in comparison to self-reported screen time measures, which are susceptible to methodological challenges.^21^

Second, many studies have been plagued by omitted variable bias. Given their limited control over pre-existing and potentially concurrent factors, cross-sectional designs, representing the majority of studies on risks associated with adolescent screen media exposure, fail to properly isolate the distinct contributions of screen exposure and private access.^21^,^22^ Therefore, a study using a prospective-longitudinal design on bedroom screen access in early adolescence that would consider competing explanations for associations promises better confound control than cross-sectional designs.^21^-^22^

Third, because girls and boys uniquely experience risk and protective factors due to distinct biological and contextual influences, sex-stratified analysis represents a more revealing approach in comparison to controlling for sex. It allows us to highlight later gender-based differences in academic and social adjustment in relation to earlier private access experience, and can stimulate our understanding of the dynamics of such differences.^23^

Using data from the Quebec Longitudinal Study of Child Development (QLSCD; described later) birth cohort, we examined the association between bedroom screen access in later childhood and academic and social adjustment in the high school senior year. More specifically, we aimed to examine whether having a bedroom television or computer at age 12years predicts subsequent self-reported academic and social adjustment by age 17years. We controlled for pre-existing individual and family characteristics that could confound these prospective associations, especially overall screen media use at age 12. Boys and girls were treated separately in our analyses. We expected that having a screen-free bedroom would subsequently predict indicators of academic and social flourishment. 

## Methods

***Participants***

The QLSCD* is coordinated by the Institut de la statistique du Qubec, and originates from a randomly selected, stratified sample of 2817 infants born between 1997 and 1998 in Quebec, Canada. The main objective of the QLSCD was to provide data on typical development in children. 

Children were selected using the province’s birth register. Of the original selection, 697 children were deemed ineligible for one of the following reasons: being a twin; having First Nations status; being untraceable at the time, mostly due to incorrect contact details; refusing to participate. The baseline sample, representing 75% of the eligible target population, comprised 2120 infants followed up annually from age 5 months throughout childhood. Of these, 39% were firstborn children. 

For each follow-up, informed consent was obtained from parents, teachers and children when applicable. Participants were included in this study if they had completed child reports on having a bedroom television and computer in the 2010 survey, when they were aged 12 years (n=1347 out of 2120). Predictor variable data were collected for 661 girls and 686 boys, thus creating our subsample for analysis. Outcome variables, based on quality and availability, were measured at age 17years.

***Measures***

**Predictor variable: early adolescence bedroom television and/or computer (age 12 years)**

Participants were asked, in two distinct questions, if they had (1) a television and (2) a computer in their bedroom. Exploratory analysis revealed similar effects of both variables on psychosocial outcomes. Therefore, we combined answers to both questions to create a variable for which 0=no to both questions (54.2 valid %), and 1=yes to having a television and/or a computer in the bedroom (45.8 valid%). 

**Outcomes variables: academic achievement and positive relations indicators (age 17years)**

*Overall grades*

In the spring of the school year, participants were asked their overall average in all school subjects. This was a discrete variable, for which participant answers ranged from 0 to 100.

*Dropout risk*

We used a variable based on the typology of dropout risk as described by Fitzpatrick et al.,^19^ comprising seven items; a higher score corresponds to a higher risk of dropping out. Items assessed school delay, performance and engagement: (1) During this school year, what is your average mark in English Language?; (2) During this school year, what is your average mark in mathematics?; (3) Have you ever repeated an entire school year?; (4) Do you like school?; (5) In terms of your school marks, how would you rate yourself compared with other students of your age at your school?; (6) How important is it for you to get good marks?; and (7)Based on your own wishes, how far do you plan to go in school?

The original variable also includes a specific category for youth who did not attend school in the past school year (based on the age of the youth in the cohort, they could not be more advanced than the fifth and final year of high school in Quebec). We created from this variable one with 3categories: 0=below median; 1=above median; 2=actual dropout (did not attend school that year).

*Prosocial behaviour*

Results of the following seven items were summed: (1) When someone got hurt, I didn’t hesitate to help them; (2) When someone made a mistake, I felt sorry for them; (3) When I witnessed an argument, I tried to stop it; (4) When someone spilled or broke something, I offered to help clean it up; (5) I helped people around me when they were having difficulty; (6) I readily shared my belongings with others; (7) I was kind to younger children. Each item explored the tendency to kindness, empathy, sharing and caring. Sums were then recoded to show a score from 0 to 10. Higher scores meant more reported prosocial behaviour.

*Recent dating relationship*

For this variable, we used answers to only one question; participants self-reported whether they had had at least one boyfriend or girlfriend in the past 12 months (0=yes; 1 =no).

**Childhood individual and family control variables (ages 5 months to 12 years, risk category = 1, no risk = 0)**

“Individual characteristics” included temperament problems, early neurocognitive skills and self-reported screen time. 

Temperament problems were assessed at age 1.5 years, reported by parents answering six questions regarding difficult and unpredictable temperament: (1) How easy or difficult is it for you to calm or appease [first name] when he/she is upset?; (2) On average, how many times per day does [first name] become restless and irritable, whether for a short or a long time?; (3) In general, to what extent does he/she cry or fidget?; (4) How easily is he/she upset?; (5) How changeable is [first name]’s mood?; (6) Please rate the general degree of difficulty that [first name] may present for the average parent. These six items were summed (above median = 1).

Early neurocognitive skills were evaluated at age 2 years, using an imitation sorting task that assesses attention and working memory, and is predictive of later academic achievement (below median = 1).^19^

Self-reported screen time was assessed at age 12 years, using weekly hours of television, internet, computer and video game exposure (above median = 1). 

“Family background characteristics” included six measures. Maternal education was assessed when the child was aged 5 months (high school diploma or less = 1). Self-reported maternal depressive symptoms were assessed at age 5 months and scored on an abridged version of the Center for Epidemiologic Studies Depression Scale (13 items; above median = 1).^24^ Parental antisocial behaviour during adolescence and adulthood was assessed at age 5 months using a composite score from mother and father responses to the National Institute of Mental Health Diagnostic Interview Schedule (higher scores correspond to more parental antisocial behaviour and correlate with social and occupational impairment; above median = 1). Parent-reported family dysfunction was assessed at age 1.5 years, using nine items from the McMaster Family Assessment Device (lower scores reveal that a family is functional; above median = 1).^25^ Family configuration was assessed at age 2 years (nuclear = 0, non-nuclear = 1). Finally, family income was also assessed at age 2 years using Statistics Canada’s low-income cut-off of that year (0 = not low income, 1 = low income). 

***Data analysis***

In this study, we examined long-term prospective linearassociations using ordinary least squares multiple regression in SPSS Statistics, version 26 (IBM Corp., Armonk, NY, US), stratified by sex. Indicators of academic achievement and positive relations at age 17 years were regressed on having a bedroom television and/or computer at age 12 years. To reduce the possibility of omitted variable bias and competing explanations, we controlled for pre-existing and concurrent child and family characteristics that could influence the predictor or outcome variables. As with any longitudinal study, incomplete data required an attrition analysis to compare the participants with varying incomplete data on control variables to participants with complete data on control variables from our sample.

With SPSS, using a stochastic algorithm, incomplete observations were imputed based on available complete data on control and outcome variables, generating multiple imputed datasets that are copies of the original complete data. The algorithm generates slightly different values for each imputed measure across the multiple datasets. The additional variance caused by differences in imputed values between the various copies reflects the uncertainty of the imputation and is added as a correction to the imputation. Our analyses were conducted with five imputed datasets, as is generally recommended.^26^

*Data compiled from the final master file ‘E1-E20’ from the Quebec Longitudinal Study of Child Development (1998–2017), Gouvernement du Qubec, Institut de la statistique du Qubec.

## Results

Table 1 provides descriptive statistics for the predictor and all outcomes and control variables. Almost half of boys and girls, at age 12, had a television or a computer in their bedroom, or both. Thirty-nine percent of boys and 43.7% of girls were born to mothers with a high school diploma or less education. More than one-fifth of the sample (21.1% for boys and 22.8% for girls) lived in a non-nuclear family by age 2, and, at the same age, more than 15% (16.3% of boys and 19.8% of girls) were from low-income families. 

**Table 1:** Descriptive statistics for predictor, outcomes and control variables

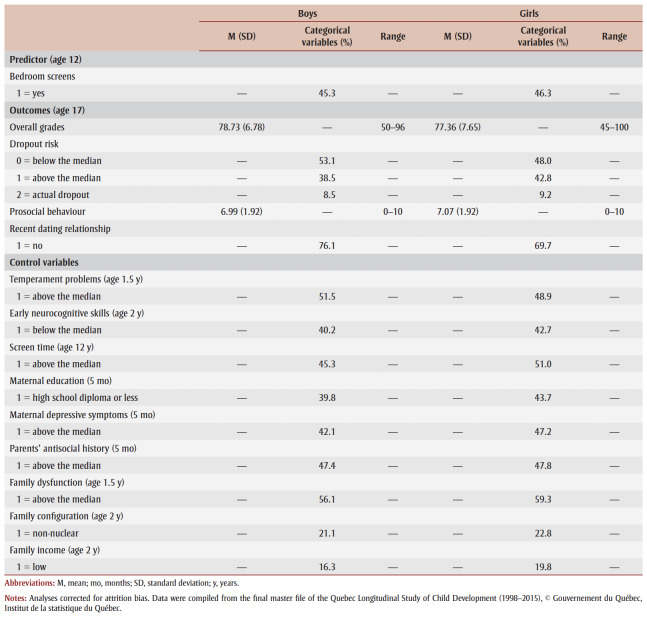

As for the outcome variables, all measured at age 17, the average grades were in the upper seventies for both boys (78.73%) and girls (77.36%). Fewer boys (38.5% above median and 8.5% actual dropouts) than girls (42.8% above median and 9.2% actual dropouts) were in the risk categories for the dropout variable. Average scores for prosocial behaviour were lower for boys (6.99) than for girls (7.07), and more boys (76.1%) than girls (69.7%) declared they had not been in any dating relationship in the past 12months. 

Table 2 documents the relationship between the pre-existing controls and having a bedroom television and/or computer at age 12 years. For boys, only maternal education when the child was aged 5 months (*B* =0.15, *p*≤0.001) predicted a higher probability of having a screen in the bedroom at age 12 years. For girls, higher levels of temperament problems at age 1.5years (*B*=0.08, *p*≤0.05) predicted a higher probability of having a bedroom screen at age 12 years. Also, having a mother who did not have more than a high school diploma when the child was aged 5 months (*B*=0.16, *p*≤0.001) and who showed higher levels of depressive symptoms (*B*=0.10, *p*≤0.01) predicted a higher probability of having a television and/or computer in the bedroom at age 12years. Non-nuclear family configuration (*B*=0.11, *p*≤0.05) also predicted a higher probability of having a bedroom screen at age 12 years for girls.

**Table 2:** Unstandardized regression coefficients (with standard errors) reflecting the adjusted relationship between baseline child and family characteristics between ages 5 months and 2 years and having a bedroom television and/or computer at age 12

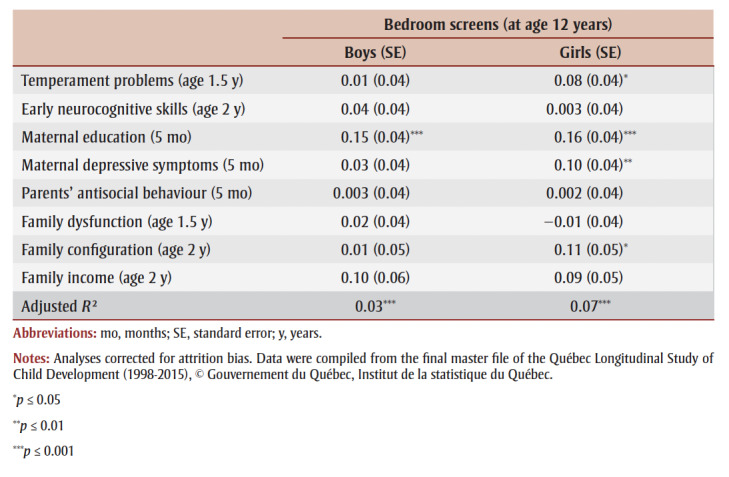

Table 3 reports the relationship between having a bedroom screen in late childhood and subsequent academic and social indicators at the end of adolescence. For boys, having a bedroom television and/or computer at age 12 years predicted lower average grades (*B*=−2.41, *p*≤0.001), higher dropout risks (*B*=0.16, *p*≤0.001), lower levels of prosocial behaviour (*B* = −0.52, *p*≤0.001) and lower chances of declaring having been in a dating relationship in the past 12 months (*B* = −0.13, *p*≤0.001) at age 17 years.For girls, it predicted lower average grades (*B* = −1.61, *p*≤0.05), higher dropout risks (*B*=0.17, *p*≤0.001) and lower chances of declaring having been in a dating relationship in the past 12 months (*B*=−0.18, *p*≤0.001) at age 17 years. 

**Table 3:** Unstandardized regression coefficients (with standard errors) reflecting the adjusted relationship between having a bedroom television and/or computer at age 12 (including concurrent screen time at age 12) and well-being indicators at age 17

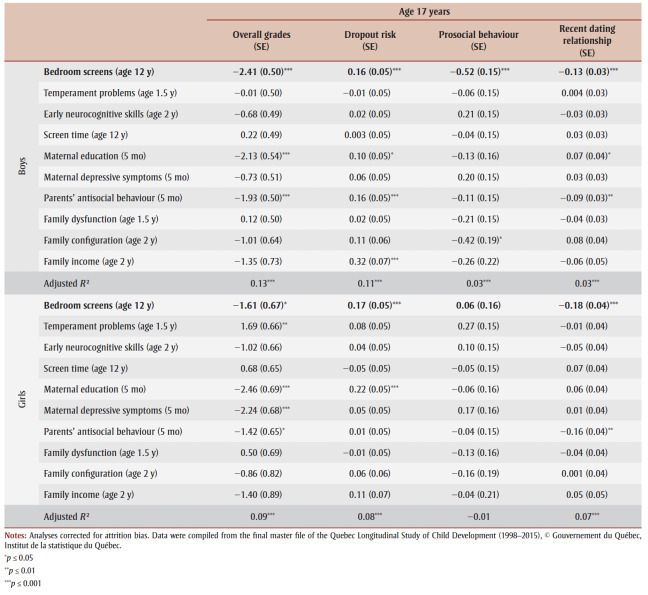

## Discussion

In the past decade, portable devices have invaded homes, making screen media more available than ever. In this context, pediatric societies have stated that there should be screen-free zones in homes, especially in bedrooms.^2^,^3^ In our sample, almost half of boys and girls had a television or computer in their bedroom when they were aged 12 years, in 2009/10. This was prospectively associated with academic and social impairment five years later. Our study suggests that private access to screens in childhood forecasts lower human and social capital by the end of adolescence. This can have notable risks associated with access and control over health and wealth in later adulthood.^20^,^27^

More specifically, by age 17 years and compared with adolescents without bedroom screens at age 12 years, private access predicted decreases in self-reported overall grades, increases in dropout risk and lower likelihood of having dating experiences in the past 12 months. We also observed decreases in propensity toward kindness, empathy, sharing and caring behaviours among boys with bedroom screens. Considering that these findings were adjusted for potential individual and family confounders, the effect sizes can be considered clinically important. In fact, the relationships observed between bedroom screens and outcomes, five years later, mattered as much or more than those with our control variables, including maternal education, a well-known early-life factor for social trajectories across childhood and adolescence.^27^,^28^ Such differences in experiences could point toward short- and long-term differences in psychosocial adjustment and well-being.^29^

Engaging in a dating relationship represents a typical developmental milestone of adolescence, which forecasts the ability to build intimate and serious relationships later in life.^30^,^31^ Adolescence is also a sensitive period for the development of prosocial skills, which contribute to overall psychological stability. Our findings on the risks associated with bedroom screens on levels of prosocial behaviour for boys are therefore compelling. This association could forecast relationships of lesser quality and lower wages in later adulthood for boys with private screen access.^11^ For girls, compared with their same-sex counterparts without bedroom screens at age 12years, we found no relationship between bedroom screen access and kindness, empathy, sharing and caring characteristics. This may be due to neurobiological modelling factors, or to societal expectations for raising daughters, who tend therefore to be more focussed on empathy and caregiving.^32^,^33^

Our findings suggest bedroom screen access in childhood poses risks later on for important developmental milestones at a time when school readiness prior to postsecondary school transition is a concern. Unfettered and unsupervised access to screens may create a time debt for academic responsibilities and nonvirtual social interactions at a time when youth are typically honing their cognitive and interpersonal skills.^7^,^15^,^18^ Such access could jeopardize the prospects of a successful life course for both sexes.^34^,^35^ Lack of face-to-face contact combined with social isolation could potentially harm adolescent development and mental health.^36^

Remarkably, self-reported screen time at age 12 years was not associated with later youth outcomes. This suggests that it is not so much the time reported spent on screens, but more the private and unsupervised nature of screen access that predicts youth outcomes in this study.^37^ We know as well that almost all discretionary screen time, for most children and adolescents, such as that which occurs in the bedroom, is devoted to recreational uses, and that very little is devoted to learning and school work.^5^

***Strengths and limitations***

The prospective-longitudinal design represents a chief strength of this study.^38^ Repeated measures with population-based cohorts are akin to conducting a natural experiment of lifestyle habits on subsequent youth outcomes. In addition, controlling for potential confounders diminishes some bias from pre-existing influences on youth outcomes. Lastly, the gender-sensitive considerations of experiences by adolescent boys and girls represent another important strength of this study.

Using secondary data analysis, our study is not without limitations. First, its nonexperimental nature precludes any causal inferences. Nevertheless, we have partially remedied this limitation by controlling for pre-existing individual and family confounding factors. Second, our database did not provide information on portable devices such as tablets and smartphones, which have proliferated in homes in recent years, and which further facilitate private access. But this is also a strength, precisely because our study takes into account unsupervised access at a time when fixed devices facilitated the measurement of this dimension.

## Conclusion

Our study supports recommendations to discourage screens from private spaces, given the associated academic and social risks. Opportunities to connect socially, interact with others and gain social competence—which are thwarted by solitary and sedentary time spent in private spaces in front of screens during adolescence—figure among the main components of optimal development and flourishment in emerging adulthood.^17^ When projected over a lifespan and across an entire population, deficits in key development factors could translate into a general propensity for costly social, economic and health problems.^4^,^39^,^40^ For these evidence-based reasons, pediatric guidelines should be more resolute about bedrooms, and other private spaces, remaining screen-free zones, especially at a time when portable devices are multiplying in homes, which may further enhance the propensity for solitary use. Limiting “anytime, anywhere” access to portable devices and mobile data before mid-adolescence could also be something for parents and policy makers to consider. Future studies, using data on smartphones and tablets, should replicate these findings during childhood and later developmental periods.

## Conflicts of interest

None. The study sponsors had no role in study design; the collection, analysis or interpretation of data; the writing; the report; or the decision to submit the paper for publication. 

***Funding***

These original sponsors funded the larger public dataset that constitutes the original Quebec Longitudinal Study of Child Development: the Fondation Lucie et Andr Chagnon, the Institut de la statistique du Qubec, the Ministre de l’ducation, the Ministre de l’Enseignement suprieur, the Ministre de la Famille, the Institut de recherche Robert-Sauv en sant et en scurit du travail, the Centre hospitalier universitaire Sainte-Justine, and the Ministre de la Sant et des Services sociaux du Qubec. 

## Authors’ contributions and statement

BG—conceptualization, data curation, format analysis, writing—original draft. 

BG, LSP—methodology, writing—review & editing. 

LSP—funding acquisition, validation. 

The content and views expressed in this article are those of the authors and do not necessarily reflect those of the Government of Canada.
